# Role of the endocannabinoid system in the pathophysiology of endometriosis and therapeutic implications

**DOI:** 10.1186/s42238-022-00163-8

**Published:** 2022-10-07

**Authors:** Harshavardhan Lingegowda, Bailey J. Williams, Katherine G. Spiess, Danielle J. Sisnett, Alan E. Lomax, Madhuri Koti, Chandrakant Tayade

**Affiliations:** 1 Department of Biomedical and Molecular Sciences, Queen’s University, Kingston, ON K7L 3N6 Canada; 2 Gastrointestinal Disease Research Unit (GIDRU), Queen’s University, Kingston, ON Canada; 3 Department of Obstetrics and Gynecology, Kingston General Hospital, Kingston, ON Canada; 4 Division of Cancer Biology and Genetics, Queen’s University, Kingston, ON Canada

**Keywords:** Endometriosis, Endocannabinoids, Infertility, Inflammation, Hyperalgesia, Phytocannabinoids

## Abstract

Endometriosis patients experience debilitating chronic pain, and the first-line treatment is ineffective at managing symptoms. Although surgical removal of the lesions provides temporary relief, more than 50% of the patients experience disease recurrence. Despite being a leading cause of hysterectomy, endometriosis lacks satisfactory treatments and a cure. Another challenge is the poor understanding of disease pathophysiology which adds to the delays in diagnosis and overall compromised quality of life. Endometriosis patients are in dire need of an effective therapeutic strategy that is both economical and effective in managing symptoms, while fertility is unaffected. Endocannabinoids and phytocannabinoids possess anti-inflammatory, anti-nociceptive, and anti-proliferative properties that may prove beneficial for endometriosis management, given that inflammation, vascularization, and pain are hallmark features of endometriosis. Endocannabinoids are a complex network of molecules that play a central role in physiological processes including homeostasis and tissue repair, but endocannabinoids have also been associated in the pathophysiology of several chronic inflammatory diseases including endometriosis and cancers. The lack of satisfactory treatment options combined with the recent legalization of recreational cannabinoids in some parts of the world has led to a rise in self-management strategies including the use of cannabinoids for endometriosis-related pain and other symptoms. In this review, we provide a comprehensive overview of endocannabinoids with a focus on their potential roles in the pathophysiology of endometriosis. We further provide evidence-driven perspectives on the current state of knowledge on endometriosis-associated pain, inflammation, and therapeutic avenues exploiting the endocannabinoid system for its management.

## Introduction

Endometriosis (EMS) is an inflammatory condition characterized by the abnormal growth of endometrial-like tissue outside of the uterus, mainly in the peritoneal cavity (Matarese et al. 2003). The condition is estimated to be prevalent in 10% of women of reproductive age (Viganò et al. 2004). The disease is heterogenous in presentation, not only for symptoms but also the lesion types ranging from superficial to deep infiltrating lesions and is associated with severe pelvic pain as well as infertility (Zondervan et al. 2018; Giudice 2010). Although the etiology of EMS is not entirely known, there are several theories for the pathogenesis of endometriotic lesions, such as retrograde menstruation, metaplasia, immune dysfunction, and stem cells (extensively reviewed here (Sourial et al. 2014; Laganà et al. 2019; Symons et al. 2018)). Sampson’s theory of retrograde menstruation is one of the oldest theories that is debated to be the root cause of EMS, given that retrograde menstruation is common among menstruating individuals (Zondervan et al. 2018; Halme et al. 1984). This theory postulates that endometrial tissue shed during menstruation is refluxed into the peritoneal cavity via the fallopian tubes, where it implants and develops as EMS lesions (Sampson 1927). Sampson’s theory, however, does not explain cases in those who do not menstruate and in cases of endometriotic lesions outside of the peritoneal cavity. It is plausible that retrograde menstruation combined with hormonal imbalance, genetic and epigenetic modifications, immune dysfunction, and environmental factors contribute to the complex pathogenesis of EMS (Barbosa et al. 2011; Montgomery et al. 2008; Bellelis et al. 2011). This complexity in pathophysiology and associated symptoms likely contribute to the difficulty of diagnosis and treatment of EMS. Currently, diagnosis of EMS is achieved through laparoscopy which has an average diagnostic delay of 7 to 11 years from the onset of the disease (Nnoaham et al. 2011). EMS patients do not have access to a treatment option that manages both pain and lesion growth, while leaving fertility intact and hence management of EMS often involves a multidisciplinary approach through a combination of nonsteroidal anti-inflammatory drugs, hormone treatment, and/or surgical excision of lesions. Clearly, this hormone-targeted therapy poses a challenge for individuals trying to conceive. One of the major goals of EMS research is to elucidate the mechanisms of lesion establishment and survival so we can develop a new generation of therapeutics to circumvent some of the challenges. Another goal is to find an ideal treatment that will not only eliminate EMS lesions but also prevent their recurrence and do so with minimal side effects (Tanaka et al. 2020).

In recent years, the endocannabinoid system (ECS) has become a topic of great interest in the field of EMS. The expanding legalization of recreational cannabinoids has led to a recent surge in the use of cannabinoids as a form of self-management therapy for many diseases, including EMS (Carrubba et al. 2021; Sinclair et al. 2019). Several articles in the past have thoroughly discussed the use and effectiveness of cannabinoids for pain management in EMS patients and the broad role of ECS in reproductive disorders (Mistry et al. 2022; Bouaziz et al. 2017; Maia et al. 2020). One of the recent meta-analyses conducted by Mistry et al. reviewed the effects of cannabis-based products on female reproductive health in the context of EMS and chronic pelvic pain and suggested that fertility complications and long-term cognitive functions might be affected. Additionally, the authors also acknowledge the lack of thorough and reliable evidence to support or dismiss the use of cannabinoids to treat EMS symptoms (Mistry et al. 2022). Similarly, a study conducted by Bouaziz et al. in 2017 summarized the role of ECS in EMS-associated pain symptoms and the potential use of cannabinoids as therapeutic agents, where they concluded that pain mechanisms are heterogenous in EMS patients and the use of cannabinoids for the treatment of EMS needs to be evaluated carefully (Bouaziz et al. 2017). The focus of this review is on the involvement of ECS with nociceptive pain, neuropathic pain, and inflammatory pain and they elegantly summarized the mechanisms of peripheral and central sensitization leading to pain amplification and psychological effects (Bouaziz et al. 2017). Even though similarities exist in some of the narrative, in this review, we specifically focus our efforts on the role of ECS in the female reproductive system in general, and we summarize current knowledge about its involvement in the pathophysiology of EMS. We address emerging literature on ECS in endometriosis lesion microenvironment and their influence on inflammation, proliferation, and vascularization. Finally, we provide insights into the potential utility of cannabinoid therapeutics in EMS.

## The endocannabinoid system

**Fig. 1:**
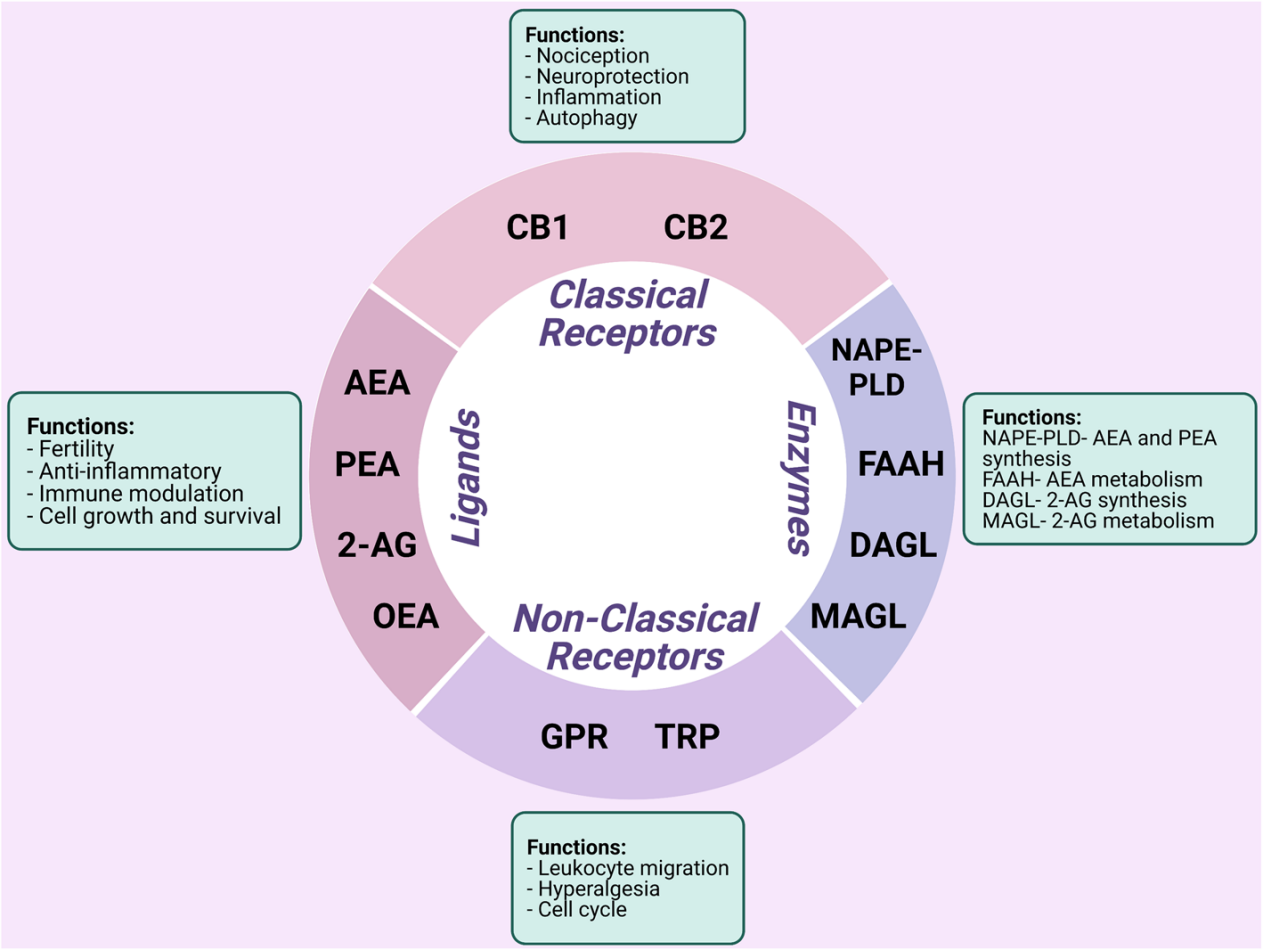
Summary of the role of the endocannabinoid system in the context of endometriosis. Cannabinoid receptors 1 (CB1) and 2 (CB2) are considered as classical receptors and orphan G protein-coupled receptors (GPRs) and transient receptor potential (TRP) channels as nonclassical receptors of the endocannabinoid system (ECS). Arachidonoylethanolamine (AEA) and 2-arachidonoylglycerol (2-AG) are the predominant molecules of the ECS, while palmitoylethanolamide (PEA) and oleoylethanolamide (OEA) are found in a lesser extent. Molecules of the ECS are biosynthesized by, but not limited to, N-acylphosphatidylethanolamine phospholipase D (NAPE-PLD) and diacylglycerol lipase (DAGL) depending on the microenvironment. Degradation of the endocannabinoids (ECs) by fatty acid amide hydrolase (FAAH) and monoacylglycerol lipase (MAGL) is rapid. Together, the ECS is involved in a variety of physiological processes such as nociception, inflammation, and immune modulation

The ECS is a ubiquitous cell signaling system that appeared early in evolution and has important regulatory and protective functions throughout the body including immune response and cell to cell communication (de Fonseca et al. 2005). The ECS consists of a complex network of enzymes, intercellular mediators, and receptors (Fig. 1). ECS receptors mainly consist of classical receptors such as cannabinoid receptors 1 (CB1) and 2 (CB2) and nonclassical receptors such as orphan G protein-coupled receptors (GPRs) and transient receptor potential channels (TRP) (di Marzo et al. 2004). Arachidonoyl ethanolamide (AEA), 2-arachidonoyl glycerol (2-AG), palmitoylethanolamide (PEA), and oleoylethanolamide (OEA) are some of the endocannabinoid (EC) ligands (di Blasio et al. 2012; Lu and MacKie 2016). EC ligands are tissue specific and are also found in circulation, stimulating both classical and nonclassical cannabinoid receptors. The ECS was first discovered in 1992, with AEA being the first EC to be isolated (Devane et al. 1992). ECs bind to the same receptors as Δ^9^-tetrahydrocannabinol (Δ^9^-THC), the active biological component of *Cannabis sativa*, which possess psychoactive effects. AEA is predominantly produced via the cleavage of its precursor *N*-acyl phosphatidylethanolamine (NAPE) by NAPE phospholipase D (NAPE-PLD) (Okamoto et al. 2004). 2-AG is synthesized by the conversion of diacylglycerol (DAG) by diacylglycerol lipase (DAGL) (Bisogno et al. 1997). The biological effects of AEA and 2-AG are believed to be terminated by cellular uptake via a putative EC membrane transporter (EMT), although it has been argued that cellular uptake occurs via membrane diffusion and could differ from cell to cell (Fowler 2013). Inside cells, AEA is metabolized to arachidonic acid and ethanolamine via fatty acid amide hydrolase (FAAH) and 2-AG is metabolized by monoacylglycerol lipase (MAGL) and FAAH to arachidonic acid and glycerol (Cravatt et al. 1996; di Marzo et al. 1998; Goparaju et al. 1999; Blankman et al. 2007). These ECs are released on demand from lipid precursors in a receptor-dependent manner immediately after their synthesis, although some have suggested that ECs can be stored in adiposomes allowing for cellular accumulation, but this remains controversial (di Marzo et al. 2004; Fezza et al. 2014). In synapse, these ECs travel in a retrograde fashion upon release to modulate activity in presynaptic cells through receptor binding (de Fonseca et al. 2005). Unutilized ECs are rapidly removed and taken up by cells where they are metabolized via enzymatic hydrolysis by the enzymes of ECS (FAAH and MAGL), suggesting that these ECs only exert effects for short periods of time before degradation (di Marzo et al. 2004).

AEA and 2-AG bind the G-coupled protein receptors such as CB1 and CB2. CB1 receptors are expressed in various regions throughout the central nervous system (CNS) including areas involved in motor activity, cognition, and sensory perception, as well as in other major tissues in the body such as the reproductive system (Venance et al. 2004). CB2 receptors are expressed on both circulating cells and tissues, as well as on some of the immune cells (Pertwee 1997; Galiègue et al. 1995). Although literature generally points out that the CB1 receptor is predominantly expressed in CNS and the CB2 receptor in the cells of the immune system, recent evidence and our own observation indicate that both receptors can be found in various systems in the body and not as restricted as reported (Howlett and Abood 2017; Lingegowda et al. 2021a). This is particularly important to take into consideration as we are aiming to either stimulate or block CB receptors for therapeutic exploitation. ECs also activate other receptors such as TRPV1 and TRPA1 receptors. TRPV1 and TRPA1 are structurally related cation channels (Huang et al. 2002)*.* TRPV1 receptors are of interest as they are involved in pain and inflammation and are activated by heat, low pH, and endogenous lipid molecules including AEA (Palazzo et al. 2008). ECs also activate the peroxisome proliferator receptors (PPAR) (Sun et al. 2006), GPR55, and GPR119, which results in the direct activation of extracellular signal-regulated kinase (ERK) and p38 mitogen-activated kinase and/or indirect activation of nuclear factor kappa B (NFkB), cyclic adenosine monophosphate (cAMP) response element-binding protein (CREB), and transcription factor 2 (ATF2) by calcium release (Syed et al. 2012; Lauckner et al. 2008).

**Fig. 2:**
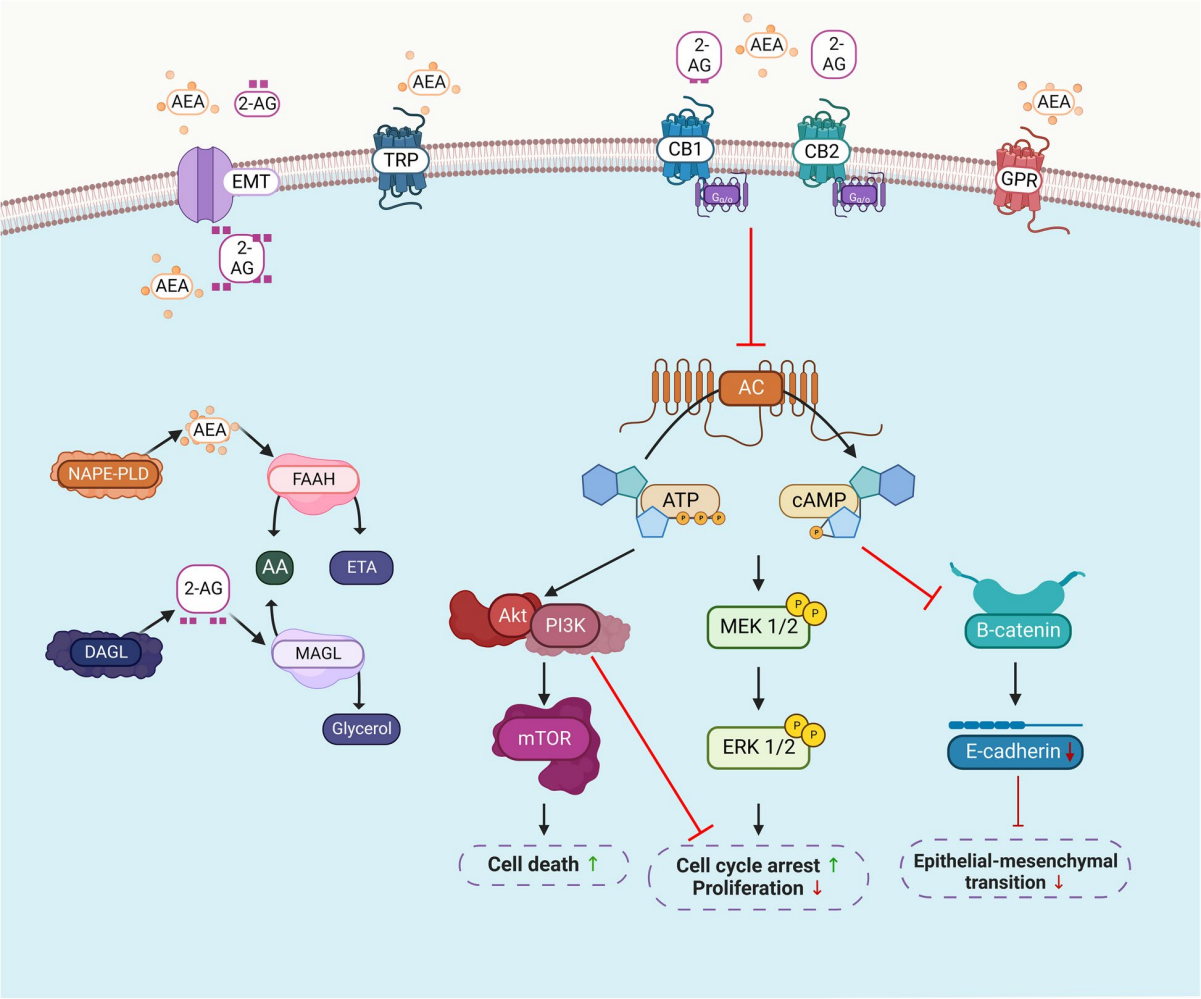
The endocannabinoid system signaling cascade. Arachidonoylethanolamine (AEA) and 2-arachidonoylglycerol (2-AG) are biosynthesized by N-acylphosphatidylethanolamine phospholipase D (NAPE-PLD) and diacylglycerol lipase (DAGL), respectively. Fatty acid amide hydrolase (FAAH) and MAGL are the metabolizing enzymes that degrade AEA to produce arachidonic acid (AA) and ethanolamine (ETA), and 2-AG to AA and glycerol. AEA and 2-AG are transported in and out of a cell through the putative endocannabinoid membrane transporters (EMTs). AEA and 2-AG bind to G protein-coupled receptors (GRPs), such as cannabinoid receptors 1 (CB1) and 2 (CB2), at varying affinities and to a lesser extent with the orphan GPR and transient receptor potential (TRP) channels. The endocannabinoid system (ECS) mainly targets the protein kinase A (PKA) signaling cascade via inhibition of adenylyl cyclase (AC)-cyclic adenosine monophosphate (AMP) that has direct inhibitory effects on β-catenin which affects epithelial-mesenchymal transition. Activation of the mitogen-activated protein kinase signaling cascades, such as extracellular signal-regulated kinase ½ (ERK ½), protein kinase B or Akt, phosphatidylinositol-3-kinase (PI3K), mitogen-activated protein kinase (MEK1/2), and mammalian target of rapamycin (mTOR), is involved in cellular processes such as autophagy, apoptosis, cell cycle, and proliferation

Endogenous and phytocannabinoids stimulate CB1 and CB2 receptors, activating multiple signal transduction pathways in cells through the G_i/o_ family of G proteins (Howlett et al. 2002). The free G_i_α proteins that are triggered upon stimulation suppress adenylyl cyclase activity and ultimately lead to the inhibition of cAMP production, which in turn reduces cAMP-regulated protein kinase A (PKA). This results in a decrease of phosphorylation by PKA that in turn modulates signaling pathways (Fig. 2). The free G_i_*β*/*γ* dimers that are triggered upon stimulation are involved in the regulation of ion channels, mitogen-activated protein kinase (MAPK), and phosphatidylinositol-3-kinase (PI3K) pathways (Howlett et al. 2002). The stimulation of the MAPK pathway upon CB1 receptor activation (through the actions of free G_i_*β*/*γ*) is the mechanism by which cannabinoids affect synaptic plasticity, cell migration, and possibly neuronal growth (Howlett et al. 2002) via calcium channels (Mackie and Hille 1992; Caulfield and Brown 1992) and potassium channels (Deadwyler et al. 1995). Conversely, the CB2 receptors did not show any modulation with either calcium or potassium channels (Felder et al. 1995).

## The endocannabinoid system and the inflammatory response in endometriosis

The ECS plays a significant role in the modulation of inflammation. The mechanisms of dysregulation in inflammatory disorders are not well known, but differences in EC levels have been observed in inflammatory compared to non-inflammatory states. As previously mentioned, the CB2 receptors are found throughout the immune system including on B lymphocytes, natural killer cells, macrophages, monocytes, microglia, and T lymphocytes, making these receptors the primary component of the ECS involved in inflammation (Cabral and Griffin-Thomas 2009; Crowe et al. 2014). Having said that, CB2 receptor knockout mice do not show gross morphological differences compared to their wild type counterparts but alteration in B cell and T cell numbers has been reported (Buckley 2008). Compensatory functions from other GPCRs make it challenging to tease out the specific effect of CB2 receptor deletion. During inflammation, macrophages are recruited to the site of inflammation through the release of pro-inflammatory cytokines and chemokines and this results in a high number of inflammatory cells at the site of trauma accompanied by edema and hyperalgesia.

The CB2 receptor agonist GW405833 inhibited inflammation through the reduction of pro-inflammatory cytokine production, such as IL-1*β* and TNF-*α*,and oxidative stress in a rat model of acute inflammation (Parlar et al. 2018). Since EMS is an estrogen-dependent inflammatory disease with increased proliferation and vascularization of the EMS lesions, the role of ECs in EMS-associated inflammation is a crucial area of interest. ECS expression, specifically through CB2 receptors on mast cells, is involved in endometrial inflammation having both inflammatory and anti-inflammatory effects based on the microenvironment (Iuvone et al. 2008). These contrasting roles show the duality of the ECS in inflammatory conditions. CB2 receptor signaling affects dendritic cell (DC) migration through the inhibition of matrix metalloproteinase-9 (MMP-9), a chemoattractant primarily secreted by macrophages and DCs which plays an essential role in immune cell migration (Adhikary et al. 2012). MMP-9 produced by DCs ensures DC migration towards inflammatory stimuli, further propagating inflammation. Indeed, MMP-9 was significantly higher in the plasma and eutopic/ectopic tissues of EMS patients as compared to healthy individuals supporting the link between CB2 receptor signaling and MMP9 (Collette et al. 2006; Liu et al. 2015). Collectively, these findings partially address the argument that the ECS is dysregulated in EMS lesions, since normal CB2 receptor signaling reduces the secretion of MMP-9 from DCs. This reduced MMP-9 activation leads to a decrease in DC migration to inflammatory sites and decreased production of pro-inflammatory cytokines such as TNF*α*, IL-6, IL-2, and IFN-*γ* in vitro (Adhikary et al. 2012). Endometriosis lesion microenvironment is highly complex with overlapping immune, endocrine alterations between stromal, epithelial, and immune cells. New approaches such as single-cell RNA sequencing of individual cell type will reveal distinct transcriptional regulation and its impact on shaping the lesion microenvironment.

One of the key mediators of lesion proliferation and survival is the MAPK signaling cascade, which is associated with macrophages during inflammatory states. During homeostasis, the MAPK signaling cascade regulates cellular processes such as proliferation, differentiation, and apoptosis through either the regulation of transcriptional factors or by direct interaction with immune mediators (Wei and Liu 2002). The ECS has been extensively studied to understand its role in modulating these MAPK family proteins and how this system responds to disease. Previous studies have identified that EC signaling is involved in the activation of the MAPK cascade, including direct activation of the p38 kinase, ERK1/2 to regulate cell cycle and growth (Wartmann et al. 1995). However, the interaction of the ECs and the MAPK cascade is stimulus dependent, where the microenvironment of the signaling cascade could either contribute to a pro-inflammatory or an anti-inflammatory response (Demuth and Molleman 2006). These MAPK family proteins are active and highly expressed in an inflammatory response such as oxidative stress, heat shock, and apoptosis (Rajashekhar et al. 2011). This is particularly important in EMS, as lesion proliferation and chronic inflammation are key hallmarks of the disease, and the activity of MAPK family proteins is significantly higher in EMS (Cakmak et al. 2018). We and others have shown that stimulation of cannabinoid receptors using a synthetic cannabinoid (WIN 55,212-2) in endometriotic cells (in vitro) directly attenuates the MAPK signaling cascade, further reducing inflammation and proliferation in these cells (Lingegowda et al. 2021b; Leconte et al. 2010).

Anti-inflammatory and analgesic effects are the two most sought-after properties of ECS, as EMS patients generally suffer from both inflammatory pain and neuropathic pain. The link between ECS and pain in EMS has been extensively reviewed by Bouaziz et al. in 2017 (Bouaziz et al. 2017). In a mouse model of inflammatory pain, the synthetic non-selective CB1/CB2 receptor agonist WIN 55,212-2 significantly reduced lipopolysaccharide (LPS)-associated inflammatory pain, regulated by the inhibition of FAAH (the chief catabolic enzyme regulating AEA). FAAH knockout mice and FAAH inhibitors had a similar effect in the LPS mouse model of inflammatory pain, both resulting in reduced edema and hot-plate hyperalgesia through CB1 and CB2 receptors. The reduction of inflammatory pain was mainly attributed to diminished pro-inflammatory cytokines, such as IL-1β and TNFα, in LPS-treated paws. These findings suggest the potential utility of FAAH inhibitors, for EC-related treatment of chronic inflammatory pain (Naidu et al. 2010). However, the specificity of these FAAH inhibitors needs to be carefully evaluated to determine whether the anti-inflammatory effects are due to selective inhibition of known pro-inflammatory markers such as IL-1β and TNFα (induced predominantly by LPS) or due to broader immune modulation.

In a zymosan-induced rat model of arthritis, CB1 receptor antagonism with AM251 appeared to contribute to inflammatory effects, while electroacupuncture led to an anti-inflammatory response through CB1 receptor-dependent activity (Gondim et al. 2012). In addition, CB1 receptor-deficient mice have a higher susceptibility to inflammatory conditions such as experimental autoimmune encephalomyelitis, a model of multiple sclerosis (Maresz et al. 2007). While new studies are shedding light on the utility of selective activation or inhibition of CB receptors, the advantage of using highly selective CB2 receptor agonists in inflammatory conditions is their ability to reduce inflammatory pain without altering basal nociception or eliciting overt psychomimetic side effects, which are often seen following treatment with CB1 receptor agonists (Kinsey et al. 2011). Although promising, it is difficult to specifically target CB2 receptors due to the structural homology between the two receptors, limiting the development of novel CB2 receptor-selective agonists (Crowe et al. 2014).

## The endocannabinoid system and pain

The analgesic effects of plant-derived or phytocannabinoids are well recorded throughout history, but we have only just begun to manipulate endogenous cannabinoids (the body’s natural pain killers). Pain relief remains a key area of research in EMS and novel analgesic treatments are continuously being investigated. Various components of the ECS are identified throughout peripheral nerve terminals (on both pre- and post-synaptic neurons), such as neurons in the dorsal root ganglia (DRG) and trigeminal ganglia and extending up to supraspinal sites that comprise nociceptive pathways (Hohmann 2002). Emerging research points out the therapeutic utility of DRG stimulation in select neuropathic pain scenarios (Berger et al. 2021; Liem et al. 2016). As previously mentioned, CB1 receptors are found throughout the CNS and can be specifically localized in regions involved in pain transmission including the spinal dorsal horn and periaqueductal gray (Herkenham et al. 1991; Tsou et al. 1998). This localization of components throughout the nociceptive system makes the ECS an attractive target for analgesic treatments.

The ECS has been well-characterized in inflammatory pain. Mice with cutaneous chemical damage displayed reduced nociceptive behavior upon administration with AEA and subsequent interaction with CB1 receptors. When treated with CB1/CB2 antagonists, mice displayed both prolonged and enhanced nociceptive behavior because of tissue damage (Calignano et al. 1998). Comparable results are reported with 2-AG through the stimulation of CB2 receptors (Guindon et al. 2007). EMS-associated pain stems from multiple pathways such as nociception, neuropathic, and inflammatory, all of which are regulated and modulated by the ECS to a certain extent. It has been proposed that some chronic pain conditions are potentially a cause of clinical endocannabinoid deficiency (CED), where ECS signaling has been dysregulated compared to individuals without chronic pain (Russo 2016). The peritoneal microenvironment is a defining factor in the establishment and progression of EMS. It is well established that levels of inflammatory cytokines (IL-1β, IL-6, and TNFα) are significantly higher in the peritoneal fluid of EMS patients. Higher levels of these cytokines fuel hyperalgesia which is further sustained through altered expression of TRPV1 in the peritoneum of EMS patients (Rocha et al. 2011). In a mouse model of EMS, we demonstrated significantly reduced expression of TRPV1 in lumbar DRGs upon exposure to WIN 55,212-2 as compared to sham mice (without EMS-like lesions), suggesting that nociceptive pain might be modulated by DRGs whose terminals are in proximity to EMS lesions (Lingegowda et al. 2021b). Similarly, a study using a rat model of EMS also showed that the stimulation of the CB1 and CB2 receptors using WIN 55,212-2 significantly reduced abdominal nociception and vaginal hyperalgesia in a CB1 receptor-dependent manner (Dmitrieva et al. 2010). Correlations have also been drawn between the levels of ECs in the plasma and peritoneal fluid, to identify their role in EMS-associated inflammatory pain. One such study showed that the levels of 2-AG and AEA were significantly higher in the peritoneal fluid of EMS patients as compared to individuals without EMS, which was also correlated to higher abdominal pain experienced by patients. Although the authors suggest that the ECs identified in their study are involved in deepening the abdominal inflammatory pain, low sample size and unaccounted comorbidities should be considered when correlating ECS involvement in driving the inflammatory pain in the peritoneum (Andrieu et al. 2022). Nevertheless, these findings demonstrate a complex and at times contradictory role of the ECS in pain modulation and management. Given the complex regulation of endogenous production of ECS, enzymes modulating endogenous levels, specific receptor engagement, teasing out cause and effect becomes a significant challenge. Most of the reported studies therefore points out to association of ECS dysregulation with disease or inflammatory state rather than causing it. More research is warranted in this domain.

## Endocannabinoids in endometriosis: what we know so far

**Fig. 3:**
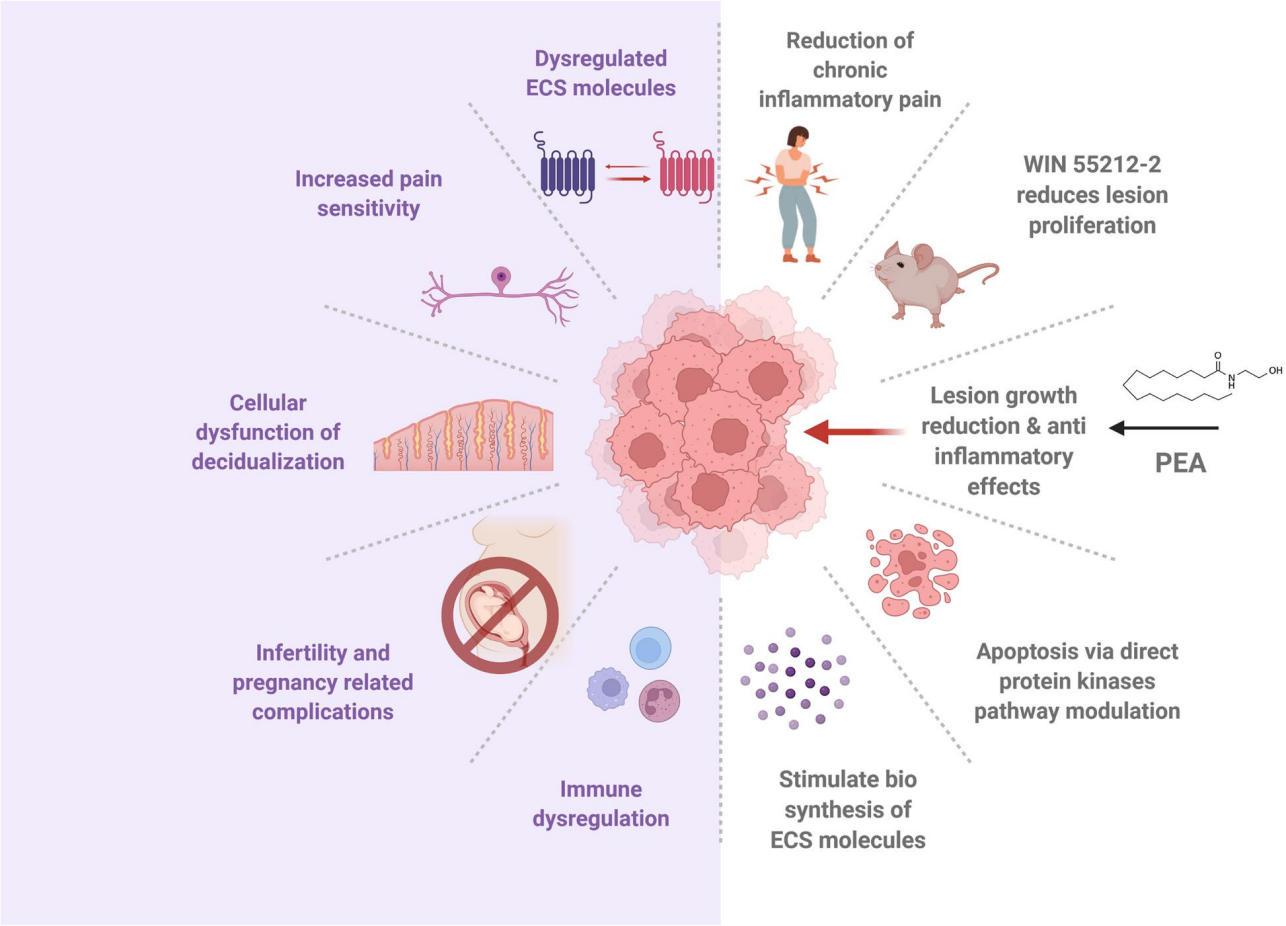
The endocannabinoid system in endometriosis pathophysiology. EMS has been associated with endocannabinoid dysregulation and deficiency that contributes to increased pain sensitivity, compromised decidualization, infertility, and related complications. Endometriotic lesions produce differential levels of endocannabinoids (ECs) but their role in disease progression versus bystander effect is not entirely known. In vivo studies have shown that synthetic cannabinoids and some ECs (palmitoylethanolamide-PEA) have anti-inflammatory effects and inhibit the proliferation of endometriosis (EMS)-like lesions in mice

EMS is characterized by pelvic pain and a dysfunctional immune response. As the ECS is involved in the regulation of both processes, it is no surprise that there are alterations in this system in those with EMS. The role of the ECS in EMS is yet to be fully elucidated, but studies have begun to show that there are differences in the ECS in patients with EMS compared to individuals without EMS (Fig. 3).

Systemically, EMS patients have elevated levels of AEA and 2-AG as well as their structural analogues, PEA and OEA as compared to women without EMS (Sanchez et al. 2016). Plasma levels of AEA, 2-AG, and OEA were also elevated in the secretory phase compared to the proliferative phase in EMS patients (Sanchez et al. 2016). Studies from our group revealed that endometriotic lesions produce members of ECS and that levels of PEA in EMS lesions were significantly higher when compared to eutopic endometrium from EMS patients (Lingegowda et al. 2021a). EMS patients presenting with moderate to severe dysmenorrhea showed higher levels of AEA and PEA when compared to those with low-to-moderate pain symptoms (Sanchez et al. 2016). These findings partly explain some of the pain symptoms in EMS as elevated levels of AEA and PEA are correlated with pain levels. Hence, several clinical trials are focused on using PEA alone or in combination with other anti-inflammatory drugs to assess the feasibility and efficacy of PEA therapy to treat EM-associated pain (reviewed elsewhere extensively) (Bouaziz et al. 2017).

Alterations in the ECS were also reported within the eutopic endometrium in patients with EMS. CB1 mRNA and protein was decreased in endometrial tissue in EMS patients compared to controls (Resuehr et al. 2012). While most of these findings on ECS dysregulation, including that from our lab, are correlative, this provides a basis that the interplay between levels of circulating and localized ECs with specific receptor expression within the EMS microenvironment may contribute to the disease pathogenesis and associated symptoms. Specific cause and effect experiments using knockout mice for each EC component and receptor will be required to address the causal role the ECS plays in EMS pathogenesis.

There are several ECs and receptor alterations in the endometriotic lesions of EMS patients. A study of adenomyosis and EMS found significantly lower CB1 and CB2 receptor expression in glandular and SCs compared to the eutopic endometrium of EMS patients and individuals without EMS (Bilgic et al. 2017). Similarly, we also showed that CB2 receptor expression in ectopic lesions was significantly lower than both eutopic endometrium from EMS patients and endometrium from individuals without EMS. The increased EC levels seen systemically may contribute to this observed decrease in CB1 and CB2 receptor expression in a negative feedback mechanism, which could impair pain modulation in this system (Sanchez et al. 2016). As signaling via these receptors typically induces anti-inflammatory effects, it can be deduced that lower levels of CB1 and CB2 receptors in endometriotic lesions contribute to a lack of anti-inflammatory effects in lesions. In contrast, other studies found equal expression of CB1 and CB2 receptors in EMS compared to controls (Leconte et al. 2010).

A decrease in FAAH, NAPE-PLD, MAGL, and DAGL enzymes has been reported in both endometriotic and adenomyotic tissues compared to healthy controls (Bilgic et al. 2017). These lower levels of catabolizing enzymes in the stromal and epithelial compartments of the lesions likely contribute to slower synthesis and degradation of AEA, resulting in higher AEA levels reported in patient plasma samples (Bilgic et al. 2017). There is also a significant elevation in TRPV1 and TRPA1 mRNA levels in ectopic endometrial tissue in patients with deep infiltrating EMS compared to autologous eutopic endometrium and healthy control endometrium (Bohonyi et al. 2017). Stromal immunoreactivities of these receptors are correlated with the severity of pain symptoms in patients, notably dysmenorrhea. These receptors are found on sensory nerve terminals and non-neuronal structures and are stimulated by pro-inflammatory molecules in the lesion environment which further triggers pain in EMS (Bohonyi et al. 2017).

EC signaling via the CB1 receptor in a mouse model has been shown to play a vital role in the symptoms associated with EMS. The sensory and sympathetic neurons that innervate and play a role in the nociceptive aspect of EMS lesions express CB1 receptors on their somata and fibers (Dmitrieva et al. 2010). A rat model exploring the impact of CB1 receptor signaling in EMS symptoms found that CB1 receptor agonists decrease EMS-associated pain, whereas CB1 receptor antagonists increase EMS-associated pain (Dmitrieva et al. 2010).

Signaling via the CB1 receptor may also contribute to the initial development of EMS lesions. Synthetic CB1 receptor agonists, such as methandamide, stimulated ESC migration through activation of PI3K and ERK1/2 pathways in a dose-dependent manner via CB1 receptors (Gentilini et al. 2010). As endometrial cell migration is a key aspect in the pathogenesis of EMS, these results suggest the potential involvement of EC signaling, more specifically CB1 receptor signaling and ERK1/2 P13K activation, in lesion establishment (Gentilini et al. 2010). Although CB1 receptor expression has been identified in EMS lesions, where neuronal innervations are observed, it is challenging to identify if CB1 expressed in the lesion (stromal and epithelial compartments) or on the innervated neurons participate in nociceptive episodes.

CB1 receptor agonists have a favorable effect on limiting cell proliferation and managing EMS-associated pain. Notably, both CB1 and CB2 receptor agonists have an anti-proliferative and pro-apoptotic effect on endometriotic SCs (Bilgic et al. 2017), suggesting that ECs increase apoptosis in EMS and offer a potential therapeutic avenue. Taken together, these findings further suggest that the ECS may play an important role in the mechanism underlying EMS pathogenesis and maintenance (Table 1).

**Table 1:** Summary of endocannabinoid (EC) molecules identified in circulation and tissues of endometriosis (EMS) patients. Most prominent ECs in circulation such as N-arachidonoylethanolamine (AEA), 2-arachidonoylglycerol (2-AG), palmitoylethanolamide (PEA), and oleoylethanolamide (OEA) were found to be altered in circulation. PEA, fatty acid amide hydrolase (FAAH), and N-acylphosphatidylethanolamine phospholipase D (NAPE-PLD) were also found to be altered in the EMS lesions, along with cannabinoid receptors 1 (CB1) and (CB2) 2 and transient receptor potential cation channel subfamily V member 1 (TRPV1)

Area	Endocannabinoid system component	Levels	Significance
Systemic	AEA/2-AG (Sanchez et al. 2016)	Elevated	Correlates with pain levels
	PEA/OEA (Sanchez et al. 2016)	Elevated	
Peritoneal fluid	AEA/2-AG^a^ (Andrieu et al. 2022)	Elevated/reduced	Inflammation
Follicular fluid	AEA (Fonseca et al. 2021)	Elevated	Inflammation
Eutopic endometrium	CB1 receptor (Resuehr et al. 2012)	Decreased	Regardless of the cycle phase
	CB1 receptor/CB2 receptor (Shen et al. 2019a)	Decreased	
	TRPV1 (Bohonyi et al. 2017)	Increased	Impact on pain
Ectopic lesion	PEA (Lingegowda et al. 2021a)	Increased	Anti-inflammatory
	CB1 receptor/CB2 receptor (Lingegowda et al. 2021a; Bilgic et al. 2017; Shen et al. 2019a)	Decreased	Inflammation
	FAAH/NAPE-PLD (Bilgic et al. 2017)	Decreased	Higher AEA levels
	TRPV1/TRPA1 (Bohonyi et al. 2017)	Increased	Increased pain
Myometrium	CB1 receptor/CB2 receptor (Shen et al. 2019b)	Increased	Correlates with pain levels

^a^During the proliferative phase of the menstrual cycle only

## Current state of cannabinoid use in endometriosis

Due to the well-established relationship between EMS and the ECS, research into the potential therapeutic effects of manipulating this system is of great interest. The use of cannabinoids (both natural and synthetic) in the treatment of EMS has varying results. It has been suggested that EC agonists are beneficial in disease profiles where stimulation of EC receptors has protective effects, such as diabetic nephropathy (Barutta et al. 2011). In EMS, this is complex as receptor activation has contradicting impacts on disease pathogenesis.

A study using palmitoylethanolamide-polydatin combination treatment of four patients reported pain relief after 1 month following treatment and patients were found to be taking fewer analgesics than before treatment. They also reported that the lesions were improving, as measured with imaging techniques (Indraccolo and Barbieri 2010). Although this study was preliminary, the impact on lesion growth aligns with literature suggesting anti-proliferative actions of cannabinoids to reduce lesion growth and manage pain associated with EMS. Similarly, another open-label study investigated the use of ultramicronized-palmitoylethanolamide (um-PEA) and co-micronized palmitoylethanolamide/polydatin m (PEA/PLD) for pelvic pain in a group of 30 EMS patients. The results showed a decrease in pain symptoms between baseline and end of treatment. Additionally, PEA treatment resulted in improved quality of life and psychological wellbeing when measured by the 36-Item Short Form Health Survey questionnaire and Symptom Check list-90 questionnaire, respectively (Loi et al. 2019).

It is evident that pain relief is a major factor that EMS patients seek in their treatment, as discussed here. Based on patient surveys and preliminary studies, some cannabinoids seem to be effective in this domain, as well as showing efficacy against EM-associated gastrointestinal, sleep, and mood symptoms (Sinclair et al. 2020; Sinclair et al. 2021a). EMS patients using cannabis have been able to reduce dosages of other medications like non-opioid and opioid analgesics, antineuropathics, antidepressants, and antianxiety medications by at least 50% (Armour et al. 2021; Sinclair et al. 2021b). It is important to note, however, that cannabinoids can have adverse effects and abuse potential, which must be considered before undergoing treatment (Volkow et al. 2014). Additional concerns about safety and legal repercussions (places where cannabis is an illegal substance) should be considered by patients. Several patients do not disclose the use of cannabis to their doctors due to legal concerns and societal judgment fears, which can lead to negative health effects as use is unregulated and unaccounted (Sinclair et al. 2021b). Some cannabinoids like Δ^9^-THC have neurological effects that can impair cognitive function. This is because Δ^9^-THC binds to CB1 receptors, present in the nervous system, where it can alter neurotransmitter release in synapses (Kendall and Yudowski 2017). Although less is known about the mechanism, CBD does not have neurological effects, which means that it could be a preferred treatment option, should it be proven to be effective. Interestingly, long-term exposure to cannabinoids in females has been shown to delay sexual maturation, cause menstrual cycle disruption, dysregulate ovarian follicular maturation, and reduce serum concentrations of luteinizing hormone and other sex hormones, making it contraindicated for those trying to conceive (Field and Tyrey 1990; Park et al. 2004; Gammon et al. 2005). Notably, AEA has become of great interest in the context of pregnancy and fertility, particularly in the elucidation of its role in pregnancy complications (Maia et al. 2020; Ezechukwu et al. 2020). The potential of manipulating the ECS therapeutically in EMS is vast but more research is needed on the exact role that ECS plays in the pathogenesis of the disease to favorably alter the system for patients with minimal side effects (Fig. 3). This is particularly relevant to EMS patients, who often suffer from infertility.

## Conclusion and future perspectives

As presented in this review, the ECS system plays an important role in both normal physiological functions and in some inflammatory disorders including EMS. EMS is a complex, hormone-dependent, inflammatory disorder where the etiology is likely multifactorial. Several molecules of the ECS such as the ligands, receptors, and enzymes are reported to be involved in the complex mechanisms of EMS both systemically and in the lesions. This observation mostly stems from dysregulated levels of ECS members in localized and systemic environments. One of the most perceived utilities of ECS is to treat EMS-associated pain; however, careful considerations are needed in targeting specific pathways since ECS is directly involved in pregnancy outcomes. Even though efforts from several researchers have contributed to the overall understanding of ECS in the context of EMS, several unanswered questions exist. Are endocannabinoids involved in endometriosis lesion proliferation, survival, inflammation, and associated pain? Are cannabinoids capable of being a mainstream therapy for EMS? Will targeting endocannabinoids affect the female reproductive system and what impact it will have on fertility and pregnancy outcomes? The current knowledge and scientific evidence available till date suggest that cannabinoid compounds have therapeutic potential for various diseases including endometriosis. However, the ECS system needs to be carefully evaluated in the future to identify (1) their direct role in the pathogenesis of EMS, (2) if ECS is exhausted given the constant inflammatory state of the lesions and hence dysregulated phenotype, and (3) selective inhibition or stimulation of ECs and their receptors for therapeutic intervention.

An ideal treatment for EMS would be to eliminate lesion, and prevention of lesion recurrence (a long-term solution), while sparing fertility with minimum side effects. Current treatments have not yet met all these expectations and disease recurrence is a major problem for patients. Although there is a major knowledge gap surrounding the ECS due to its complex network and function, the cannabis research landscape is swiftly evolving on both therapeutic fronts. Like any prescription medication, cannabinoids must also be regulated to manage daily intake before it can be considered as a prescription medication for EMS management, among other regulatory and safety requirements. Despite this need for regulation, most of the self-management strategies employed by EMS patients are currently nonspecific. One of the unexplored avenues of therapeutic potential is to boost the innate production of EC ligands such as palmitoylethanolamide (PEA), which is known to be anti-inflammatory in nature. Unfortunately, the knowledge of ECs has not yet caught up to the fast-paced pharmaceutical world and this presents a challenge for its future use in therapeutics. With the growing establishment of cannabis-dedicated research institutes, we can anticipate that the knowledge gap that exists today will be closed in the near future and provide clear ideas on the therapeutic potential of cannabinoids in EMS and other disorders.

## Data Availability

Not applicable

## Abbreviations

EMS: Endometriosis
ECS: Endocannabinoid system
EC: Endocannabinoid
CNS: Central nervous system
DRG: Dorsal root ganglia
CB1: Cannabinoid receptor 1
CB2: Cannabinoid receptor 2
GPR: G protein-coupled receptor
TRP: Transient receptor vanilloid potential channels
AEA: Arachidonoyl ethanolamide
2-AG: 2-Arachidonoyl glycerol
PEA: Palmitoylethanolamide
OEA: Oleoylethanolamide
Δ9-THC: Δ9-Tetrahydrocannabinol
NAPE-PLD: N-acyl phosphatidylethanolamine phospholipase D
DAGL: Diacylglycerol lipase
EMT: Endocannabinoid membrane transporter
FAAH: Fatty acid amide hydrolase
MAGL: Monoacylglycerol lipase
PPAR: Peroxisome proliferator receptors
ERK: Extracellular signal-regulated kinase
MAPK: Mitogen-activated protein kinase
PI3K: Phosphatidylinositol-3-kinase
MMP-9: Matrix metalloproteinase-9
COX: Cyclooxygenase
